# Modeling the positioning of single needle electrodes for the treatment of breast cancer in a clinical case

**DOI:** 10.1186/1475-925X-14-S3-S1

**Published:** 2015-08-27

**Authors:** Agnese Denzi, Lidia Strigari, Franco Di Filippo, Claudio Botti, Simona Di Filippo, Letizia Perracchio, Mattia Ronchetti, Ruggero Cadossi, Micaela Liberti

**Affiliations:** 1 Italian Inter-University Centre of Electromagnetic Fields and Bio-Systems (ICEmB), Department of Information Engineering, Electronics and Telecommunication (DIET), University of Rome "La Sapienza," Rome 00184, Italy; 2 Laboratory of Medical Physics and Expert Systems, Regina Elena National Cancer Institute, Rome 00144, Italy; 3 Department of Surgery, Regina Elena National Cancer Institute, Rome 00144, Italy; 4 Department of Pathology, Regina Elena National Cancer Institute, Rome, 00144, Italy; 5 Clinical Research, IGEA SpA, Via Parmenide 10/A, Carpi, Italy

**Keywords:** Electrochemotherapy, Breast Cancer, Single Needle Electrodes, Numerical Modeling, Electrodes Positioning

## Abstract

**Background:**

Breast cancer is the most common cancer in women worldwide and is the second most common cause of cancer death in women. Electrochemotherapy (ECT) used in early-phase clinical trials for the treatment of primary breast cancer resulted in a not complete tumor necrosis in most cases. The present study was undertaken to analyze the feasibility to use ECT to treat patients with histologically proven unifocal ductal breast cancer. In particular, results of ECT treatment in a clinical case are compared with the ones of a simplified 3D dosimetric model.

**Methods:**

This clinical study was conducted with the pulse generator Cliniporator Vitae (IGEA, Carpi, Italy). ECT procedures were performed according to ESOPE standard operating procedures. Five single needle electrodes were used with one positioned in the center of the tumor, and the other four distributed around the nodule. Histological images of the resected tumor are compared with the maps of the electric field obtained with a simplified 3D model in Comsol Multiphysics v 4.3.

**Results:**

The results of the clinical case demonstrated a reduced efficacy of the ECT treatment described. The proposed simple numerical model of the breast tumor located in a low conductive tissue suggests that this is due to the reduced electric field induced inside the tumor with such 5 electrodes placement. However, where the electric field is predicted higher than the reversible electroporation threshold (E>400 V/cm), also the histological images confirm the necrosis of the target with a good agreement between the modeled and clinical results.

**Conclusions:**

The results suggest the dependence of the effectiveness of the treatment on the careful placement of the electrodes. A detailed planned procedure for the tumor analysis after the treatment is also needed in order to better correlate the single electrode positions and the histological images. Simulation models could be used to identify better electrodes configuration in planning the experimental protocol for ECT treatment of breast tumors.

## Background

Breast cancer is the most common cancer in women worldwide. The World Health Organization [1], reported that cancer is a leading cause of death [2] and that breast cancer is the second most common cause of cancer death in women, with a 521000 deaths in 2012. Typical treatments are based on surgical procedure (lumpectomy or mastectomy), often the surgical removal is combined with radiation therapy, chemotherapy or hormone therapy as an adjuvant therapy [3]. The invasiveness of the surgical technique has lead to an interest in new types of minimally invasive treatments such as radiofrequency ablation, cryosurgery and irreversible electroporation [4-9].

In these last years, electrochemotherapy (ECT) has been evaluated as a new possible technique to treat different kind of tumors [10-13].

Electrochemotherapy (ECT) is a technique that combines chemotherapy (insertion of non permeant or low permeant chemotherapeutic drugs with high cytotoxicity) with local application of pulsed electric fields to the tumor nodule. Electric pulses induce cell membrane reversible electroporation thus increasing drug diffusion into the cell and its cytotoxicity; (see [10] for an overview). Bleomycin and Cisplatin are the common drugs used for ECT. In particular, when the Bleomycin is used for ECT its activity in vitro experiments is increased 1000-fold [14] and in vivo up to >80 times thanks to the membrane reversible electroporation.

The results of two European projects: Cliniporator™ (IGEA SpA, Italy), with the development of the medical devices needed for tissue reversible electroporation (electrodes and pulse generator), and ESOPE with the definition of the operating standard procedure for ECT [15], have permitted the adoption of ECT in routine clinical use [16].

In clinical practice, according to standard operating procedures validated in the ESOPE project, monopolar, direct current electric pulses are used. In particular, the protocol consists of 8 monopolar 100 μs pulses with a repetition frequency ranging from 1 Hz to 5 kHz. Pulses are delivered through needle or plate electrodes with a fixed geometry, the electric field is applied to each electrode couple in order to obtain an electric field in the tumor roughly equals to 400 V/cm [10]. At present, ECT is most commonly used to treat metastatic tumor nodules located at the skin and subcutaneous tissue [10,17-19].

With the aim of expanding the clinical indications of ECT to treatment of tumor nodules located deep within the body, e.g. liver metastasis, soft tissues sarcoma, etc. clinical studies using treatment planning based on numerical models, have been conducted, see [11] for an overview.

The extension of electrochemotherapy treatment to deep-seated metastases [11,20] and bone tissue [11,21,22] has been possible thanks to technological advances on new electrode type with variable geometry. In particular, for this kind of tissues, single needle rigid electrodes are used [11,20]. In [10,20,23-25] it is suggested that with the variable geometry of these electrodes, an incorrect positioning could compromise the effectiveness of ECT treatment. In particular, in [20], for the treatment of melanoma metastasis, the treatment planning was performed for two different electrodes configurations: one with four electrodes outside the tumor and the other one with a five electrodes, one in the center of the tumor and the others located just around it, suggesting that the five electrodes setup would have been preferable to the other one configuration.

In [10] is reported that ECT has been used in two independent early-phase clinical trials for the treatment of primary breast cancer at the time of diagnosis in a neoadjuvant approach [26] before tumor removal. Histological analysis at the time of surgery has demonstrated that tumor necrosis was not complete in most cases. Locally, an important inflammatory response was present and surgeons complained of the presence of fibrosis that made reconstruction difficult, both studies are currently active, but not enrolling patients while data are further analyzed. The study was conducted with the same 5 - electrodes configuration proposed in [20].

In this paper we study the feasibility to use ECT to treat patients with histologically proven unifocal ductal breast cancer. In particular, the description and analysis of the ECT treatment in a clinical case are compared with the results of a simplified 3D dosimetric model with the aim to interpret and justify the observed results.

The aim is to demonstrate how the chosen configuration is not efficient when the tumor is completely inserted in a low conductive tissue, as it was the case of the breast tumors reported in [10]. At the same time, we aim to show how it is still possible to relate the electric field distribution (electric field value higher than 400 V/cm as set in [10]) with the effectively treated area.

## Materials and methods

### Experimental protocol

The clinical study was conducted with the pulse generator Cliniporator Vitae (IGEA, Carpi, Italy). This generator permits 6 independently controlled and electrically insulated outputs each providing up to 3000 V, max current 50 A, delivering 8 rectangular electrical pulses (rise time 1 μs) of 100 μs duration [21,27]. The electrodes, made of medical grade stainless steel, have a diameter of 1.2 mm that allows an easy insertion of them inside to tumor.

Electrochemotherapy procedures were conducted according to ESOPE standard operating procedures when taking into account drug used, route of administration, dosage, and timing of electric pulse application, namely 15 U/m^2 ^intravenous belomycin was administered in a bolus 8 minutes prior to electric pulses application. Application of pulses was completed within further 20 minutes.

The electrode configuration used five electrodes, one positioned in the center of the tumor, and the other four distributed around the lesion like in [10]. The positioning of the electrodes was manually controlled. The working hypothesis was that this configuration could lead to an effective reversible electroporation of the tumor and at the same time of the surrounding tissue. Treatment of the margins is very important because it could prevent possible presence of microscopic disease or irregular shape of the tumor itself. During the operation, the Cliniporator device saves all the necessary information to reconstruct the treatment parameters in term of: electrodes and their activation, distance between the electrodes (in cm), amplitude of the applied pulses (in V), duration of the applied pulses (in μs), number of pulses, such information is shown in Table 1 for the investigated clinical case.

**Table 1:** Parameter values extracted by Cliniporator with information about the electrodes and their distance, the amplitude, duration and number of pulses.

Probe From	Probe To	Distance (cm)	Amplitude (V)	Pulse Length (μs)	Number Pulse
1	2	2	2000	100	8
1	4	2	2000	100	8
2	3	2	2000	100	8
3	4	2	2000	100	8
5	1	1.4	1400	100	8
5	2	1.4	1400	100	8
5	3	1.4	1400	100	8
5	4	1.4	1400	100	8

In this case, the patient was subjected, in sequence, to 8 pulses with duration of 100 μs and amplitude of 2000 and 1400 V for the external electrode pairs and the pairs with the center electrode respectively. The 1000 V/cm was considered as the voltage-to-distance ratio used to guide what voltage should be applied between each electrode and does not represent the discrete electric field exposure.

During the procedure, the voltage applied and the absorbed current due to each pulse are measured and stored in the data output files, from these data it is possible to extrapolate the resistance values between the couples of electrodes.

**Figure 1:**
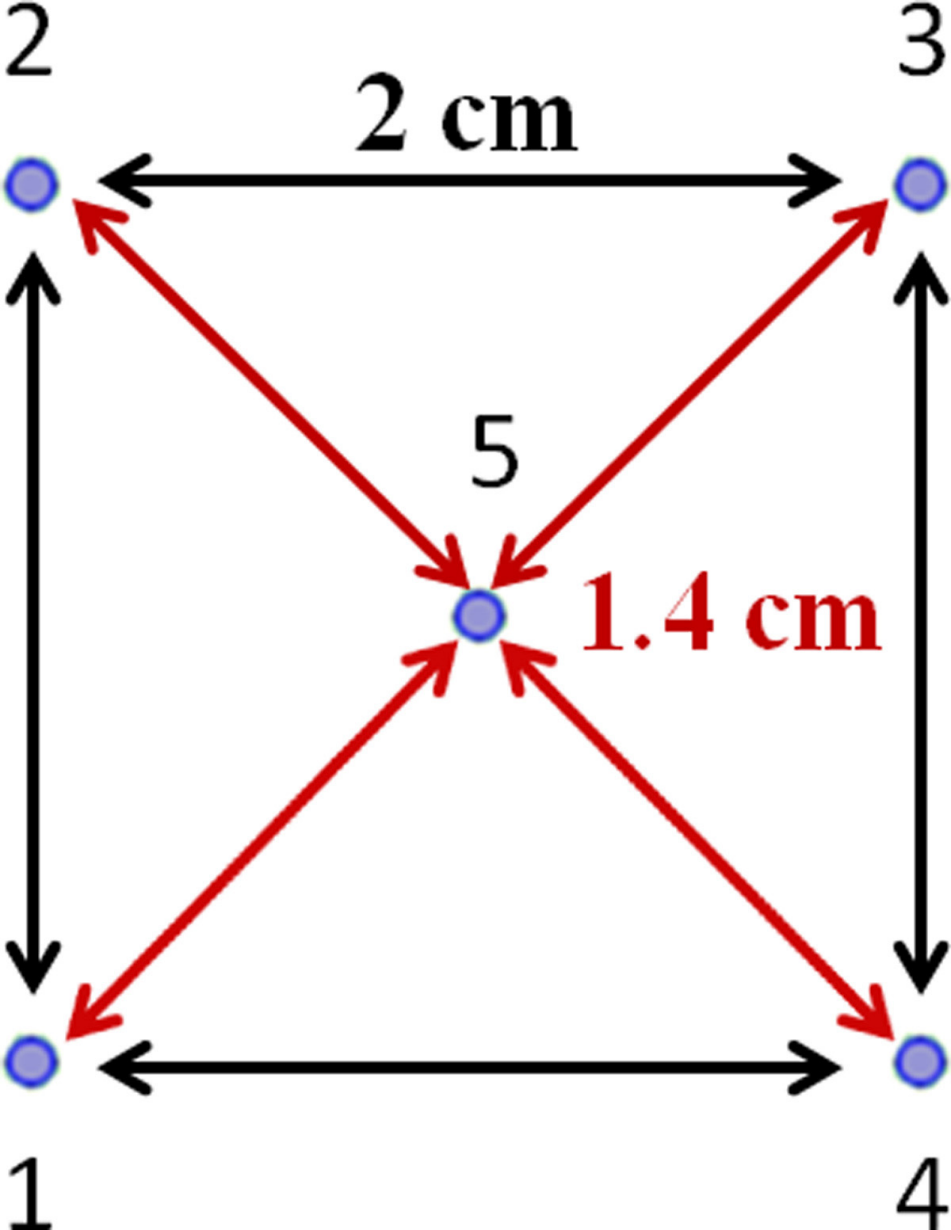
****Positioning and distance between the electrodes for the clinical case examined****.

Electrode configuration and positioning are schematically represented in Figure 1. The electrodes are numerated as in the Cliniporator file (Table 1).

### Numerical model

The proposed model is a simplified 3D reconstruction of the tumor and its environment; the target was modeled as a pseudo ellipsoid considering the dimension extracted from the histological images obtained after the treatment, the surrounding tissue was chosen homogeneous.

The model was built in Comsol Multiphysics v. 4.3; the AC/DC Electric Current physics was used in the simulation in a stationary condition, solving the Laplace equation:

∇⋅(σ∇V)=0

where E=-∇V.

**Figure 2:**
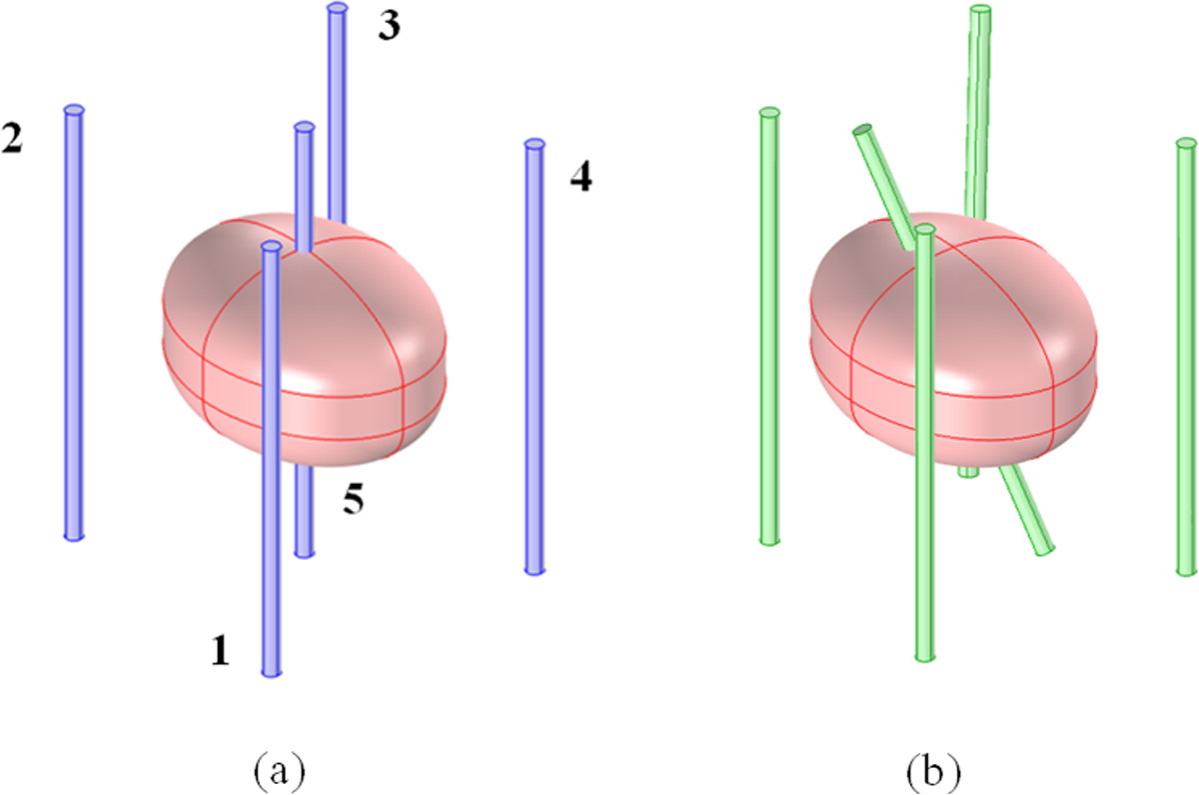
**3D model of the tumor in breast tissue treated with a 5 electrodes configuration**. The two different configurations are reported: (a) "Symmetrical" model (blue straight standing electrodes equally spaces) and (b) "Asymmetrical" model (green electrodes) the central electrode is tilted of *α *angle (25°), the external electrodes are moved with displacements along the line that connect the external with the central one of about 1.8 mm, 2.8 mm and 2.1 mm for the pairs 1-5, 2-5 and 3-5 respectively. The position for the electrode number 4 remains unchanged in the two electrode configurations.

The 3D model is reported in Figure 2.

The 5 electrodes configuration was considered with the same positioning reported in Figure 1 (active parts of the electrodes in blue in Figure 2.a) and the same stimulation parameters saved in Cliniporator's output files (Table 1): 2000 V between the external electrodes and 1400 V between each external electrodes and the central one. This was the first simulated configuration that we called "Symmetric" model.

A second model was developed in order to take into account eventual uncertainties in the position of the electrodes due to their manual insertion in and around the target, based on the information reported by the surgeon. Variations in the inter-electrode distances and rotations of the insertion directions have been considered. As reported in Figure 2.a, the electrodes in the symmetrical model are placed at distance of about 1.4 cm with respect to the central electrode. In the asymmetrical configuration (Figure 2.b green electrodes) the external electrodes 1, 2, and 3 are moved and placed at distance of about 1.22 cm, 1.12 cm, 1.19 cm with respect to the central one. In particular the displacements along the line that connect the external with the central one are about 1.8 mm, 2.8 mm and 2.1 mm for the pairs 1-5, 2-5 and 3-5 respectively. The position for the electrode number 4 remains unchanged. The central electrode is rotated with an angle α of about 25° plausible according to the surgeon that made the treatment. This second configuration was called "Asymmetric" model.

The electrodes were 1.2 mm in diameter and presented an electrode activate length of 3 cm, reflecting the physical dimensions of the ones used during the ECT treatment. In the model, electrodes are stainless steel as the real ones (σ = 4.032 × 10^6 ^S/m).

The tumor dimensions are extracted from the histological images and the information taken during the procedure: the principal axes of the pseudo ellipsoid are 2 cm × 1.5 cm with a thickness equals to 1.3 cm. The surrounding tissue was represented as a box of 10x10x10 cm^3^.

The numerical model takes into account the changes in conductivity based on electric field strength in situ as suggested in [24,25,28-31]: the tumor and the mammalian tissue conductivities were considered variable as a step function from their initial values as in [29]. The reversible electroporation threshold and the irreversible one are fixed at 400 and 800 V/cm respectively [32]. The initial values were set: for the tumor σ = 0.55 S/m, as suggested in [33-35] for the breast cancer, and for the surrounding box σ = 0.04 S/m as in [33,36,37] for mammalian tissue with high percentage of fat.

Electric insulation conditions are placed at all the boundaries of the simulation box except for one side where an impedance condition is placed in order to take into account the possible heterogeneity of the breast tissue and its interfacing with the rest of the body (σ = 0.5 S/m, [37]). The electrodes are set to potential or ground in pairs as in the used ECT protocol, and electric continuity condition is considered in the other internal boundaries.

Reproducing the experimental protocol, 8 simulations were carried out activating in sequence the 8 pairs of electrodes. The total solution was extracted in terms of maps of electric field combining the maximum values obtained among all the sequences results thanks to a routine developed with Matlab R2010a.

The results are reported in terms of maps on 2D slices at the same position of the ones extracted from the histological images in order to have a one-to-one comparison between the modeled and the clinical results.

## Results

A phase I study was planned to assess the feasibility and safety of ECT on unifocal breast cancer through the evaluation of the histological response obtained on the resected surgical specimen. Inclusion criteria were: patients with unifocal ductal breast cancer, palpable, diameter less than < 3 cm histologically proven by evaluation ER, PgR, HER-2; absence of inflammatory features; good definition of lesion to ultrasound; distance between tumor margin and skin > 0.5 cm; no metastases; written informed consent. Exclusion criteria were multifocal carcinoma; allergy to bleomycin; extended component in situ; extralesional microcalcification; pregnancy; breast-feeding; epilepsy; presence of pacemaker. The protocol has been approved by the Institutional Ethical Committee.

The enrolled patient was affected by breast cancer, located at right side at the union of the upper quadrants (dimension was about 2.0 cm × 1.5 cm × 1.3 cm).

Paraffin embedded surgical material was obtained from specimens of breast cancer patient. The tissue specimens were fixed in 10% buffered formalin, embebbed in paraffin, then sectioned at 3 millimicron and stained with Hematoxilin and Eosin. Histological type and sub classification were determined according to the Bloom Richardson mod Notthingham method, using the istoscore assigned to three features: tubules formations, nuclear pleomorphism, mitotic count, ranging from 1 to 3. The overall grade was obtained by summation of the scores for the three variables. Immunohistochemistry was performed to test the status of the oestrogen, progesterone, Ki67 index and HER2 status. The samples were attained by biopsy before ECT.

After the ECT treatment all these evaluations were performed again on the treated tissue. The second sample of tissue was obtained directly from the resected tumor at the time of tumor removal (i.e. about 1 month after ECT). The treated tissues showed different areas with marked fibrosis and entrapped foamy, histiocytes and chronic inflammatory cells, giant-cells granulomas, haemorrhagic fields, steatonecrosis. The epithelial component was damaged by cytho-architectural alterations, with nuclear anomalies and nuclear debris. Ratio viable cells/fibrosis was more or less 60-50/100. The receptors and HER2 status were the same.

**Figure 3:**
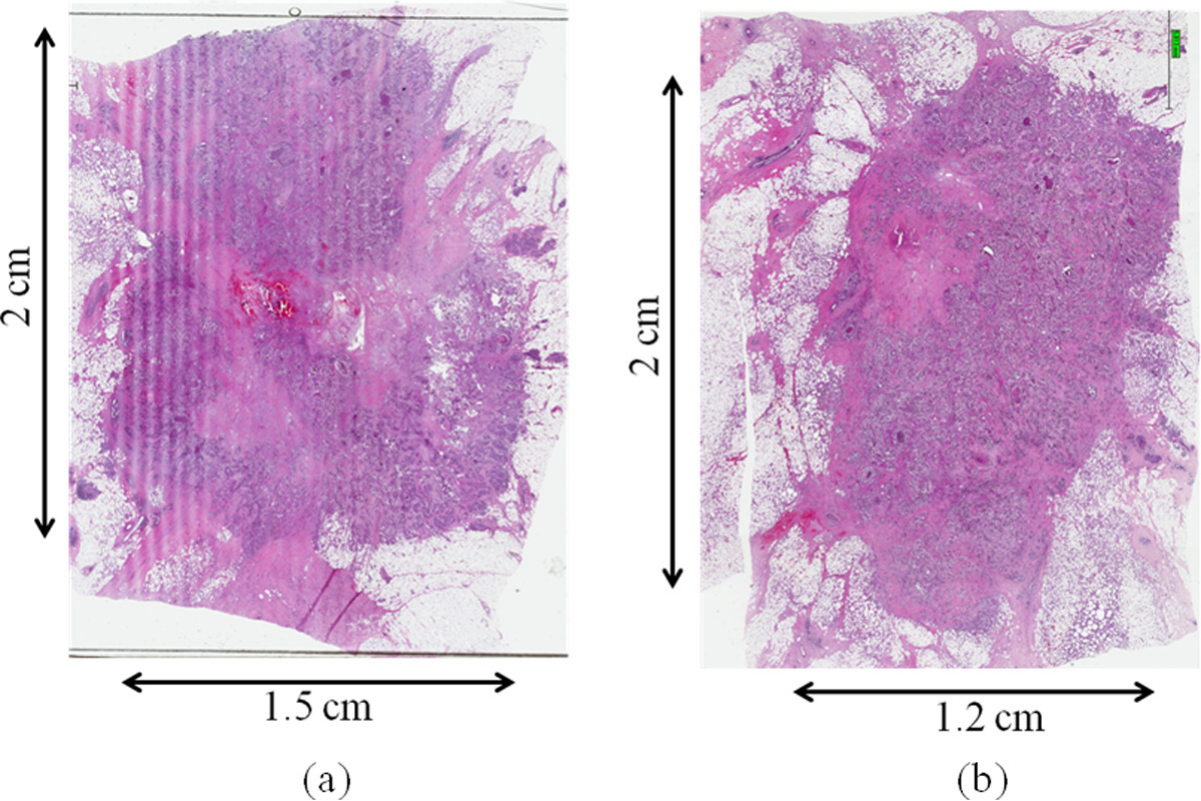
**Histological images: (a), (b) histological images of two different tumor slices after the surgical removal of the treated tumor**.

Figure 3.a and b reports the histological images of two different tumor slices after the surgical removal of the treated tumor. The shape of the tumor is well approximated by an ellipse with a principal axis of 2 cm and 1.5 cm. The thickness of the tumor is reported in the clinical files and is about 1.3 cm.

In Figure 3.a it is possible to recognize, in the center of the tumor the lesion due to the insertion of the internal electrode, whereas around it, the necrotic area has a shape of tails, more defined in the upper region of the tumor. The histological image shows an asymmetric effectively treated area.

A possible explanation of this asymmetry has been hypothesized in little inaccuracies during the manual electrodes insertion that could lead in a not perfectly balanced electrical stimulation.

**Figure 4:**
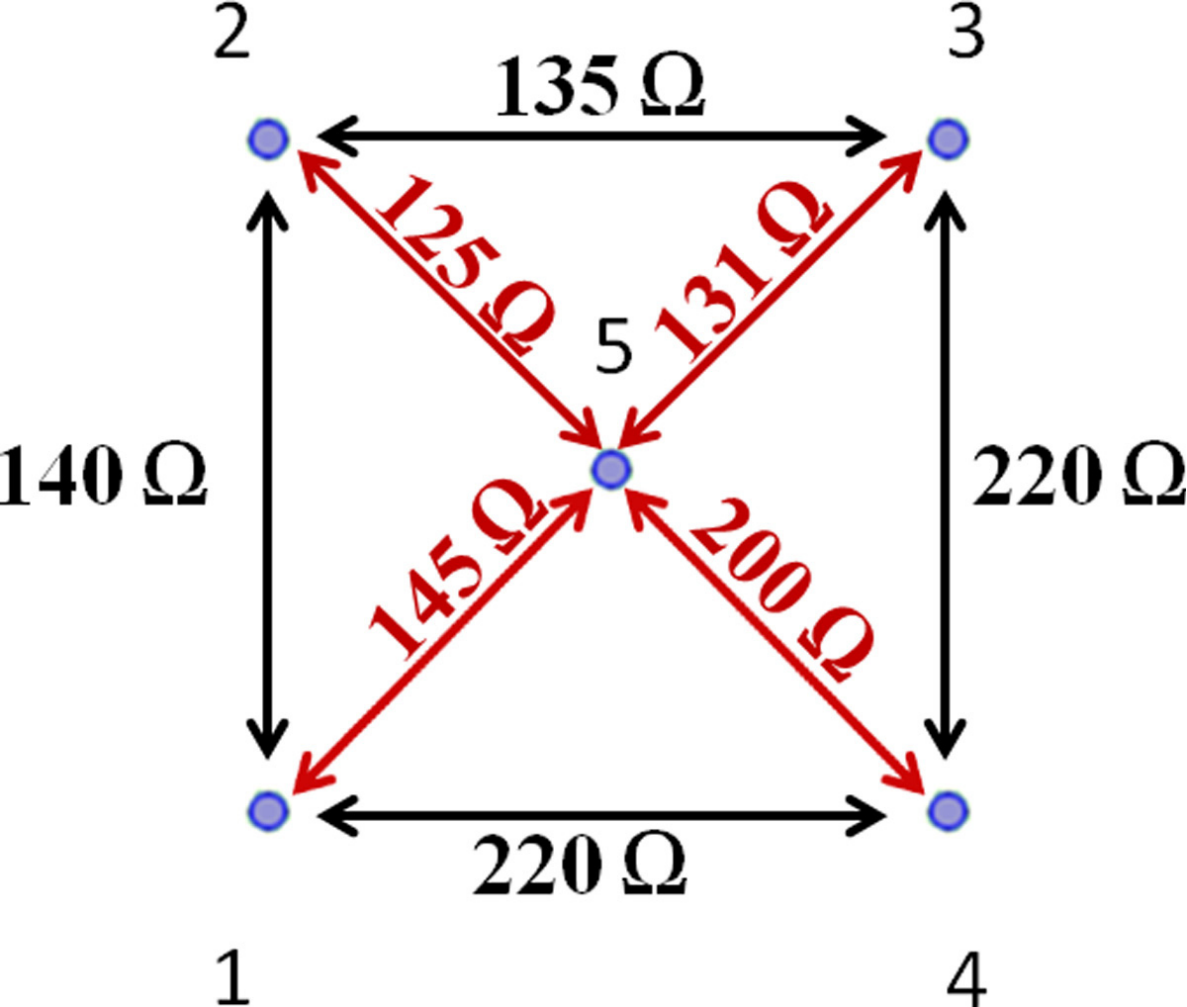
**Resistance values between each electrode pairs**.

To validate this hypothesis, the indirect measure of the resistance evaluated between each electrode pair during the treatment was considered. In particular, considering the voltage and the current measured between the electrodes during the procedure, it was possible to assess the resistance values. The results of this calculation are shown in Figure 4.

In Figure 4, we can observe a difference in the measured resistance between different pairs. Even if the clinical procedure did not note the actual electrode placing with respect to the tumor geometry, we can speculate that electrode pairs with higher resistance can be associated with the regions with lower effective results and the ones with lower resistance with the most extensive treated area. Hence, the electrode number 2, that presents the lower resistance both with its adjacent external electrodes (1 and 3) and with the central one (5), has been associated to the larger treated area (upper left tail in Figure 3.a); whereas the electrode number 4, that presents the higher value of resistance with its adjacent external electrodes (3 and 4) and with the central one (5), is associated to the region with smaller necrotic area (bottom right zone in Figure 3.a). Equivalent reasoning is carried out for the other pairs.

These experimental aspects can be interpreted with the results coming from the numerical modeling.

**Figure 5:**
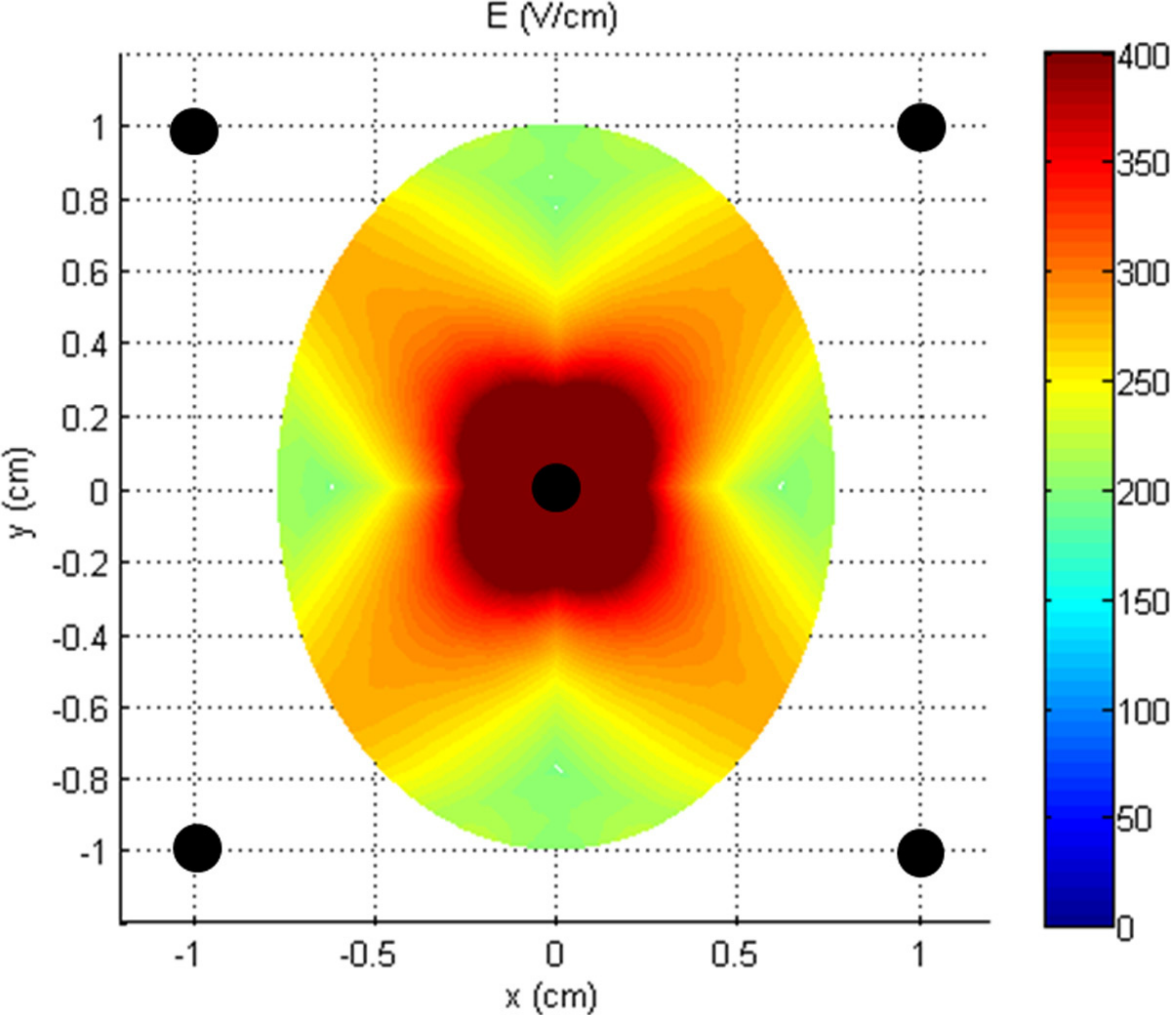
**Electric field distribution inside the tumor lesion due to the "Symmetric" model of Figure 1**.

In particular, the results obtained from the simulations with the "Symmetric" model of Figure 2.a (blue electrodes) are reported in Figure 5 in terms of electric field distribution induced in the tumor due to the whole treatment procedure (see Section Materials and Methods). It is possible to note a central region where the electric field is higher than the reversible electroporation threshold (400 V/cm) and a sort of tails similar to the ones observable in the histological images even if they are below the defined threshold. These results clearly suggest that the incomplete effectiveness (only 17 % volume of the tumor was treated) of the treatment can be related to a lack of induced electric field inside the tumor, due to the low conductance of the external tissue that prevent current density to flow inside the tumor between the internal electrode and the external one, forcing it to remain confined around the electrodes [7].

To give a tentative interpretation of the asymmetry of the results and to obtain electric field distributions that matched the histological images of the necrotic area, we considered the "Asymmetric" model of Figure 2.b (green electrodes). In particular we hypothesized, also considering the information taken by the surgeon during the procedure, that the uncertainties in the position could be modeled in the central electrode by considering an inclination of it during the insertion and in little translations of the external electrodes, suggested also from the resistance values. Indeed exact positioning cannot be guaranteed since the unguided procedure.

In Table 2 the measured and computed currents are reported for each electrodes pairs demonstrating that they are reasonably close together, considering the proposed simplified model.

**Table 2:** Measured and computed currents for each electrodes pairs.

Probe From	**Probe To**	Measured Current (A)	Computed Current (A)
1	2	14.3	14.7
1	4	9.1	14.5
2	3	14.8	15.1
3	4	9.1	14.2
5	1	9.7	11.2
5	2	11.2	11.6
5	3	10.7	11.3
5	4	7	10.7

**Figure 6:**
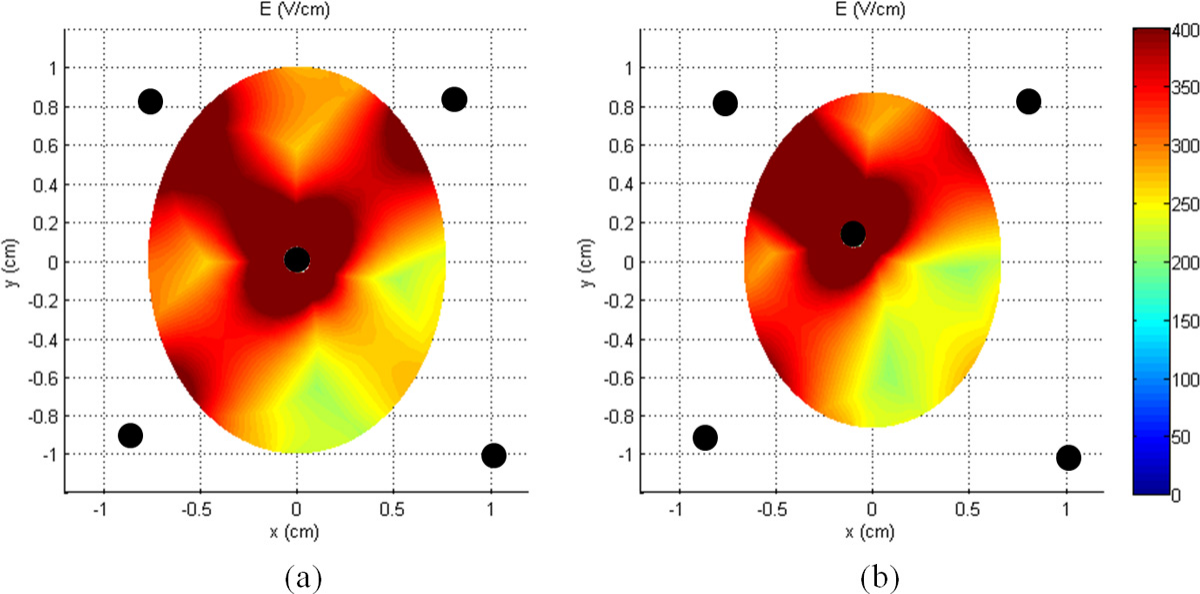
**Electric field distribution for the "Asymmetrical" model: (a) slice at z = 0 and (b) z = 4 mm**.

In Figure 6 the electric field distribution with this new asymmetrical configuration is reported with the current position of the electrodes. The asymmetry of the field on the slice taken at z = 0 (Figure 6.a) and the slice taken at z = 4 mm (Figure 6.b) is evident.

**Figure 7:**
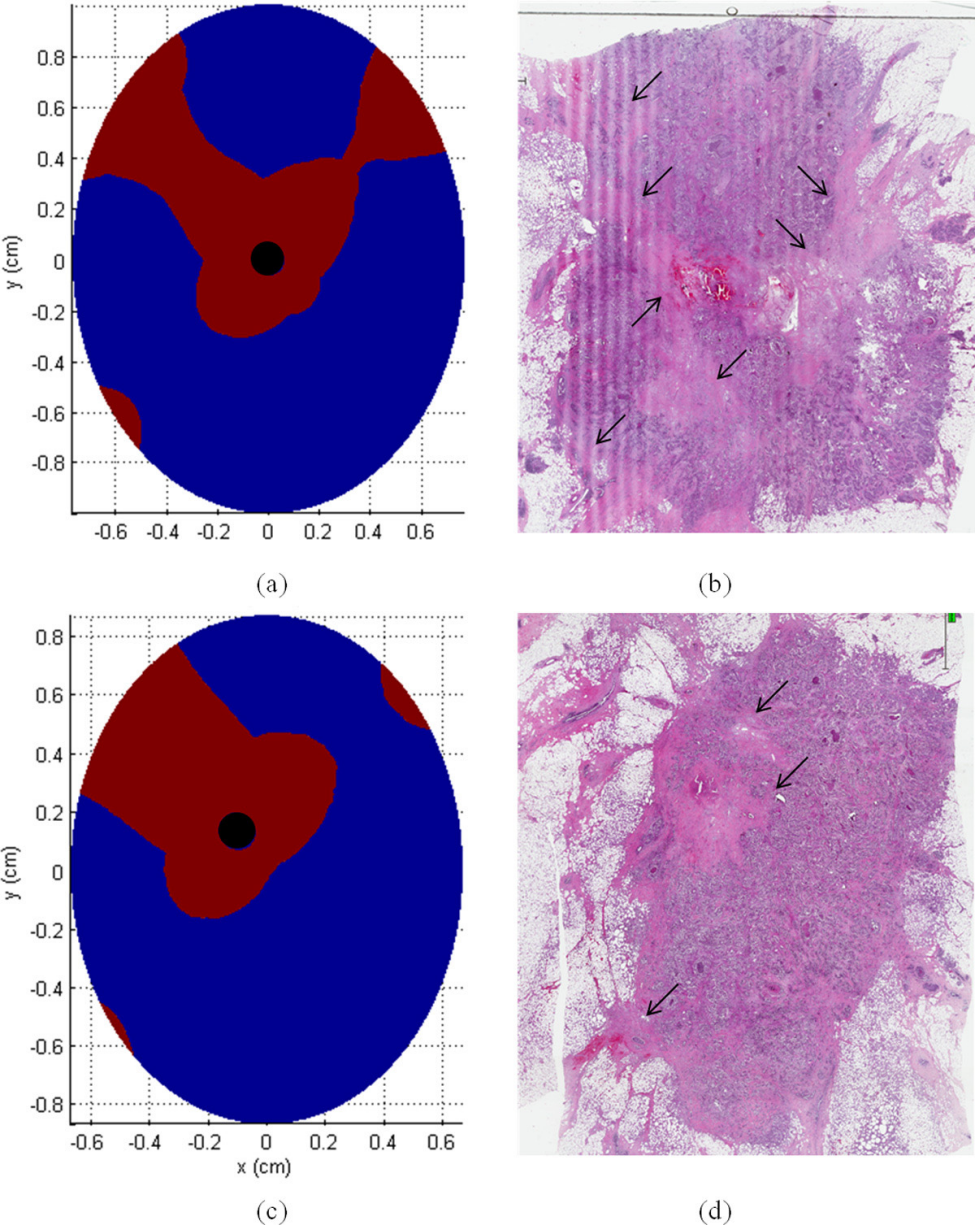
**(a) and (c) electroporated area in the tumor slices and (b) and (d) interpretation of the necrotic area in the histological images**.

In Figure 7, we can observe a good agreement between the necrotic area in the histological image (Figure 3) and the area in which the electric field overcomes the threshold for reversible electroporation E > 400 V/cm (red area in Figure 7.a and c). The asymmetry of this area on the slice taken at z = 0 mm (Figure 7.a) seems consistent with the asymmetry presented in the histological image of the tumor slice of Figure 7.b. We can observe the presence of the tails in the upper region and absence of electroporated area in the bottom right region. This result seems important in order to link the reversible electroporation effect on the tumor with the electric field distribution inside it. Figure 7.c reports the region with E > 400 V/m for the model taken at z = 4.0 mm, the result for this slice confirms the presence of an area prevalent in the portion of the tumor on the upper left zone as in distribution of the necrotic area of Figure 7.d. The volume percentage of the treated tumor is about 29 %.

## Analysis and discussion

The results of the clinical case demonstrated a reduced efficacy of the ECT treatment with the described 5 electrodes configuration when applied to breast cancer treatment. The proposed simple numerical model of the breast tumor located in a low conductive tissue suggests that this is due to the reduced electric field induced inside the tumor with this electrodes placement. This configuration not only is not efficient for the ECT treatment of the tumor but, at the same time, produced high values of current densities and electric field around the external electrodes: the field, due to the low conductive healthy surrounding tissue, remains confined outside the tumor, effectively limiting the electroporated tumor volume. The numerical results for two slices of the tumor model taken at z = 0 mm and z = 4.0 mm show that the regions where the electric field is predicted to be higher than the reversible electroporation threshold (E>400V/cm), have a similar shape of the regions of necrosis in the histological images (Figure 3 and 7.b and d). These results seem important in order to link the ECT effect on the tumor with the electric field distribution inside it, in accordance with what reported in [38]. These results clearly support the importance of pre-treatment planning and at the same time the importance of a very accurate positioning of the electrodes during the procedure in order to optimize the treatment based on predictive results as previously suggested for other cancer treatments [10,20,23-25].

**Figure 8:**
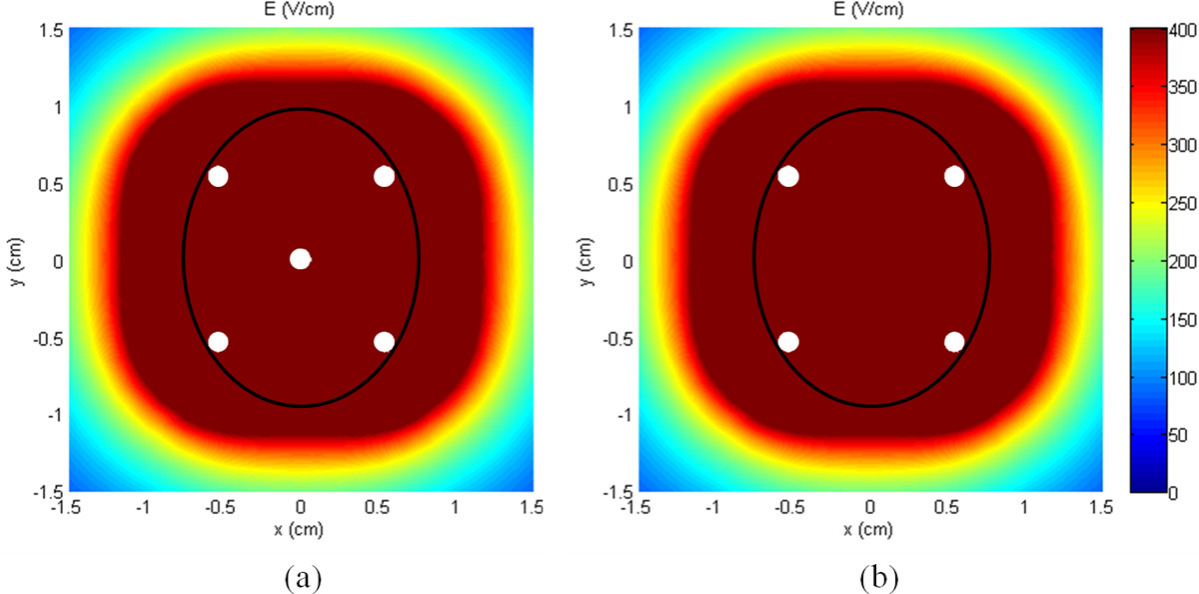
**Electric field distribution with the 5 (a) and 4 (b) electrodes inside the tumor**.

The good agreement between the clinical and numerical results, in terms of necrotic area from the histological images and area with E> 400 V/m extracted from the simulations, indicates that numerical models are able to predict the electroporation distribution and could be used, in principle, to suggest optimal electrodes configuration. As an example, Figure 8 shows that 100% of tumor volume can be electroporated in the same slices previously examined by avoiding to place the single electrodes (4 or 5) externally to the tumor, but internal to it and almost tangent to the boundary surface. This configuration demonstrates the possibility to span the electroporated area also outside the tumor as in the original intent of the chosen configuration. The data of Figure 8 does not aim to suggest a precise positioning, that should be the objective of a dedicated study, but to be the proof of principle that, in the case of tumors located inside low conductive tissue, electrode placing inside the tumor is preferable. However some issues should be deepened like the hardness of the tumor for electrode insertion or safety of the extraction procedure.

Our findings support and confirm the suggested approach for placing the electrodes on the interior margin of the tumor rather than around the periphery for conductive tumors in low-conductivity surrounding tissues as reported in [8,9] in which they demonstrate as placing the electrodes within the more conductive tumor circumvents the issues encountered for this type of heterogeneous system, and permits effective electric field distributions within the tumor.

## Conclusions

In this paper the results for a clinical case of breast cancer treated with ECT treatment were analyzed. The chosen configuration of 5 electrodes, one placed in the center of the tumor and the others 4 outside of it, resulted inefficient to obtain the complete reversible electroporation of the tumor. These results were explained using a simplified numerical 3D model of the tumor. The modeled results suggested that the inefficient treatment is due to a reduced electric field induced inside the tumor with this electrodes placement. However, when the electric field is predicted higher than the reversible electroporation threshold (E>400V/cm), also the histological images confirm the necrosis of the target with a good agreement between the modeled and clinical results. These results seem important in order to connect the ECT effect on the tumor with the electric field distribution inside it.

We can conclude that a more detailed planning of the procedure for the tumor analysis after the treatment is needed in order to be able to get a more certain correlation between the single electrodes positions and the histological images. However, the simulations clearly suggest that the effectiveness of the treatment strongly depends on the careful placement of the electrodes: moving or tilting the electrodes may lead to changes of effectiveness in the ECT treatment itself [24].

In the future an important step will be to determine accurately the conductivity of the involved tissues. Moreover simulation models could be used to identify better electrodes configuration in planning a new experimental protocol for ECT treatment of breast tumors.

## Competing interests

Mattia Ronchetti is a full time employee of IGEA S.p.A. Ruggero Cadossi is president of IGEA S.p.A. and holds company stocks.

## Authors' contributions

MR, LS and FdF developed the underlying concept of this study, with contributions from RC and ML. FdF, CB, and SdF performed the surgical interventions and ECT treatments, LS, LP carried out the analysis. AD and ML prepared the models and performed the simulations. All authors discussed results, interpreted data and formulated findings. AD and ML wrote the manuscript, with contributions from MR and LS.

## Declarations

Publication costs for this article were funded by IGEA S.p.A

This article has been published as part of *BioMedical Engineering OnLine *Volume 14 Supplement 3, 2015: Select articles from the 6th European Conference of the International Federation for Medical and Biological Engineering (MBEC 2014). The full contents of the supplement are available online at http://www.biomedical-engineering-online.com/supplements/14/S3.
